# Extreme temperatures and the epithelial barrier theory

**DOI:** 10.1007/s00281-026-01087-8

**Published:** 2026-10-20

**Authors:** Ismail Ogulur, Huseyn Babayev, Can Zeyneloglu, Bingjie Zhao, Ceren Bicer, Xiaoqing Liu, Yanni Zhang, Paolo D’Avino, Manru Li, Angel Maldonado, Jennifer Hotie, Sena Ardicli, Yagiz Pat, Mübeccel Akdis, Kari C. Nadeau, Cezmi A. Akdis

**Affiliations:** 1 Swiss Institute of Allergy and Asthma Research (SIAF), University of Zurich, Herman Burchard Str. 9, Davos, CH-7265 Switzerland; 2 Department of Biochemistry and Molecular Biology, School of Chemistry, Complutense University of Madrid, Madrid, Spain; 3 Department of Genetics, Faculty of Veterinary Medicine, Bursa Uludag University, Bursa, Turkey; 4 Department of Environmental Health, Harvard T.H. Chan School of Public Health, Boston, MA USA

**Keywords:** Barrier dysfunction, Epithelial barrier theory, Exposome, Extreme temperatures, Noncommunicable diseases

## Abstract

Extreme temperatures are now recognized as significant global health threats, driving morbidity and mortality across diverse diseases by compromising epithelial barrier integrity and causing periepithelial inflammation. The epithelial barrier theory posits that environmentally induced barrier dysfunction, driven by modern industrial and urban exposures, leads to compromised surfaces of the exposed tissues accompanied by epithelial activation and barrier dysfunction, microbial dysbiosis, loss of commensal bacteria, opportunistic pathogen colonization, bacterial translocation, inflammation and immune dysregulation. While toxic chemical exposures have been the primary focus of barrier research, temperature extremes—now intensified by anthropogenic warming, altered weather patterns and regional climatic variability—may similarly disrupt epithelial structure and function. Heat and cold can alter tight-junction regulation, induce mitochondrial and endoplasmic reticulum stress, oxidative stress, apoptosis and modify cytokine and chemokine profiles, thereby weakening barrier defenses and enhancing susceptibility to pollutants, allergens and pathogens. These effects may be amplified by co-exposures such as humidity fluctuations, UV radiation, wildfire smoke and increased pollutant loads associated with floods, droughts and other environmental catastrophes. Shifts in the exposome, including contamination of water systems and food chains, further link temperature-related barrier injury to rising rates of noncommunicable diseases (NCDs). Clinical and epidemiological evidence supports associations between heat waves and disease exacerbations, between cold exposure and infectious or allergic responses, and between altered weather patterns and growing prevalence of NCDs. This review synthesizes mechanistic, environmental and epidemiological data to highlight extreme temperatures as critical drivers of epithelial barrier dysfunction and global disease burden.

## Introduction

Extreme temperatures—both heat and cold—pose a growing threat to global health, with substantial regional disparities in mortality and morbidity. In Europe, a comprehensive analysis across 854 cities revealed 203,620 excess cold-related deaths and 20,173 excess heat-related deaths, corresponding to age-standardized rates of 129 and 13 per 100,000 person-years, respectively [1]. The findings highlight considerable regional variation and provide critical evidence for policymakers to design targeted climate and public health interventions. Modelling of 1,368 European regions further projects that under a 3 °C warming scenario, the cold-to-heat death ratio could drop from 8.3:1 to 2.6:1, with approximately 55,000 additional deaths expected by year 2100 [2].

In Japan, nationwide modelling similarly found that cold-related mortality continues to exceed heat-related mortality, while heat-related morbidity has risen markedly. The divergence between mortality and morbidity emphasizes the need for tailored interventions and adaptive healthcare planning, especially for older adults and males who face a higher risk [3].

Globally, cold spells remain a significant death risk, causing an estimated 205,932 excess deaths annually between years 2000 and 2019, with the highest burden observed in Europe. In contrast, extreme heat events since 2000 have resulted in over 260,000 deaths worldwide, as increasingly frequent breaches of “uncompensable” wet-bulb temperature thresholds threaten human survivability [4–6]. Furthermore, high temperatures contributed to around 150,000 deaths and 3.4 million disability-adjusted life years (DALYs) from noncommunicable diseases (NCDs) in 2019, predominantly in South Asia and the Middle East [7].

The effects of extreme temperatures, both heat and cold, are evident as novel occupational hazards, which implies a major daily threat in the work labor representing a huge socioeconomic burden worldwide. This is especially clear in outdoor workplaces, causing heat shocks and dehydration under heat events, as well as cold associated issues including skin injuries, cold or flu during cold events [8, 9]. Concurrently, workplace exposure to chemical irritants and particulate matters of less than 2.5 μm (PM2.5) significantly damages not only the skin barrier but also the main barriers in the body, including the airways or gut, representing a major threat that should be carefully considered [10, 11].

According to the World Health Organization (WHO), anthropogenic climate change is a major threat to human health in the modern world because it increases the frequency, intensity and distribution of environmental stressors. The accumulation of greenhouse gases such as carbon dioxide, methane and nitrous oxide in the atmosphere lead to a global rise in temperatures, increased rainfall, rising sea levels, extreme weather events, and changes in the growing seasons and land use [12–16]. All these processes are interconnected and constitute environmental stressors that have a significant impact on many aspects of humans, animals and plants. Temperature increase can be the seed of all other environmental stressors and thus has a direct effect on life, as well as an indirect impact through all the other stressors [17, 18].

Historical temperature variability provides important context for this review because human societies have repeatedly confronted periods of regional warming, cooling, drought and hydrological instability, yet the biological and public-health consequences of those episodes depended strongly on local geography, nutrition, housing, labor patterns and infectious-disease ecology. The Medieval Climate Anomaly and the Little Ice Age were not uniform global events, but heterogeneous regional intervals of warmth, cooling and altered precipitation that influenced harvest reliability, famine risk, population movement, settlement density and exposure to respiratory, waterborne and vector-associated infections [19]. These prehistoric fluctuations offer a sobering reality check for modern global warming, serving as a warning that shifting climate regimes can trigger severe habitat destruction, widespread species extinction, and rapid ecological upheavals [20, 21]. This perspective is relevant for the epithelial barrier theory because thermal stress does not act alone. Heat, cold, humidity, smoke, dust, nutrition, pathogen pressure and hygiene conditions converge on the same exposed interfaces of the skin, airways and gut. Modern anthropogenic warming differs from earlier climate variability in rate, global coherence, greenhouse gas forcing and projected persistence, which limits the usefulness of historical analogies as direct predictors. Nevertheless, historical climate shifts provide a useful conceptual model for understanding how thermal volatility can reorganize the exposome and disease risk. IPCC assessments indicate continued warming during the 21st century, with more frequent hot extremes, intensified hydrological cycles, changing precipitation, sea-level rise and regional variability that may include persistent cold-season extremes or abrupt cold exposures despite the global warming trend [22]. Therefore, epithelial barriers should be considered not only in relation to mean temperature rise, but also in relation to repeated thermal transitions, short recovery intervals, humidity shifts, regional heat and cold extremes, and compound exposures that include pollutants, allergens, microbial products and contaminated water or food systems. This broader temporal perspective supports the central argument that extreme temperatures are independent barrier-damaging stressors whose clinical impact is amplified when they coincide with nutritional stress, occupational exposure, crowding, microbial dysbiosis and environmental contamination.

A clear association is observed between a 1 °C increase in body temperature and an enhanced mortality rate [23]. Plenty of studies examining this correlation suggest that different disorders worsen with increased temperatures, such as cardiovascular diseases, heat stroke and dehydration [24–27]. A meta-analysis associated the increase in temperature with a rise in suicide outcomes (attempt or completed), of hospital attendance and of mental disorder admission [28]. Additionally, air pollution, floods, droughts and wildfires lead to worse nutrition in vulnerable populations that has direct effects on sperm quality, hormonal balance and ovulation, thus affecting reproductive health, promoting reduced fertility rate, miscarriage, stillbirth and preterm birth [29].

**Fig. 1:**
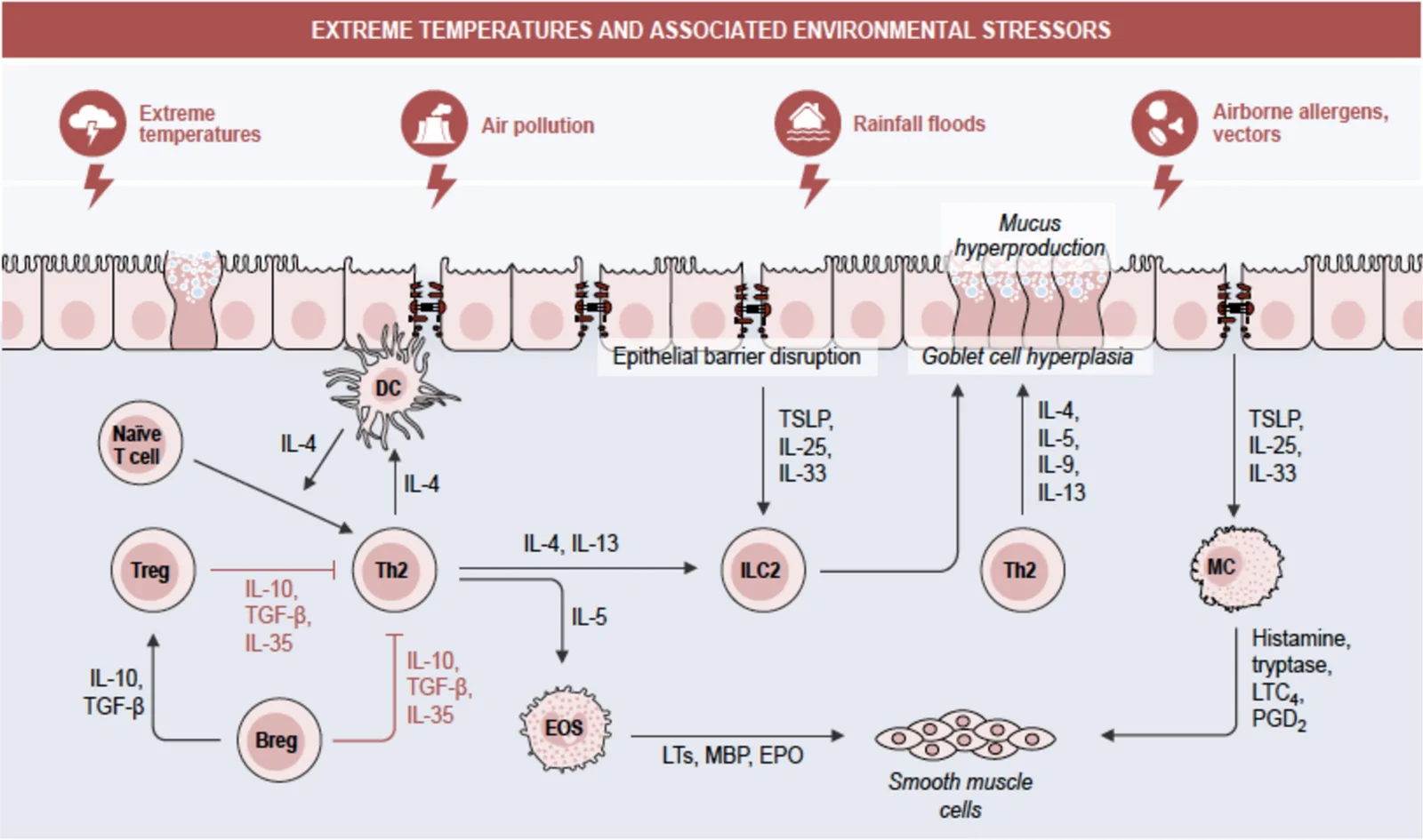
Extreme temperatures and associated environmental stressors as epithelial barrier-damaging agents. Extreme temperatures, along with other climate change features like air pollution, rainfall floods and airborne allergens or vectors, disrupt both the skin and airway epithelial barriers, thus leading to dendritic cell activation and alarmin (TSLP, IL-25 and IL-33) secretion. These events promote a type 2 immune response mediated by Th2 polarization, ILC2 and mast cell activation. The resulting feedback loop contributes to goblet cell hyperplasia, mucus hyperproduction, worsening epithelial barrier disruption as well as eosinophil recruitment and smooth muscle cell activation. Breg, regulatory B cell; DC, dendritic cell; EPO, eosinophil peroxidase; IL, interleukin; ILC, innate lymphoid cell; LT, leukotriene; MBP, major basic protein; MC, mast cell; PGD2, prostaglandin D2; TGF, transforming growth factor; Th, T helper cell; Treg, regulatory T cell; TSLP, thymic stromal lymphopoietin

Furthermore, increased temperature is associated with dysregulated immune responses characterized by a pro-inflammatory innate immune system with increased pro-inflammatory cytokine secretion (e.g., TNF-α, IL-1β, IL-4 and IL-6) and cell numbers [30]. Mechanistically, increased temperature induces oxidative stress that activates heat shock factors that promote heat shock proteins (HSP) 70 and 90 expression, which shifts the cell into a pro-inflammatory status [31]. HSPs can be protective and anti-inflammatory inside healthy cells; however, they can be pro-inflammatory when overexpressed, mislocalized, or released during stress or damage through increased readiness for inflammasome activation and inhibitory IκB inactivation and NFκB activation. Similar effects were also observed in the adaptive immune system with an increased Th2/Th1 ratio [25, 32]. In line with this observation, increased temperatures exacerbate type 2 diseases like asthma and atopic dermatitis (AD). In the context of asthma, this type 2-prone response is accompanied by a disrupted epithelial barrier, with reduced tight junctions and increased permeability and alarmin production. Phenotypically, heat exposure increases mucus production and triggers airway hyperreactivity in in vivo models and severe asthma exacerbations with increased asthma-related hospital admissions and worsening in quality of life (Fig. 1) [24, 33].

Multiple mechanisms link increased temperatures to a epithelial barrier damage as a key starting point for downstream disease-related effects. In this context, it is noteworthy to note the epithelial barrier theory provides a mechanistic framework for understanding the phenotype induced by extreme heat events [34–36]. The epithelial barrier theory aims to link chronic NCDs to environmental stressors that disrupt the epithelial barrier [34, 36]. Barrier disruption induces epithelial inflammation, alarmin release and proinflammatory cytokine and chemokine production as the key upstream processes that then may cause a systemic inflammation (Fig. 1) [37, 38]. Epithelial barrier disruption has already been associated with type 2 diseases such as asthma [39], AD [40] and inflammation [41, 42], partly through mechanisms related to oxidative stress.

Global climate change shifts long-term temperature averages and meteorological patterns, accelerating localized physical and chemical exposures [43, 44]. At the host level, extreme temperatures, such as acute heatwaves and cold spells, impose direct thermodynamic and metabolic stress on cells [45, 46], while altering weather patterns modify regional precipitation, relative humidity, and storm frequency [47]. These meteorological variations often amplify environmental stressors, including particulate air pollution, wildfire smoke, and airborne allergens, which directly contact mucosal and cutaneous tissues [48, 49]. Although global climate change drives these macro-environmental shifts, localized thermal, meteorological, and chemical exposures affect epithelial barrier integrity and function. Here, we comprehensively review the effects of extreme temperatures, both heat and cold, across different organ systems with an emphasis on the underlying cellular and molecular mechanisms of damage, adaptive responses, and implications for human health under current and projected climate scenarios. We further evaluate how thermal stress alters epithelial tight junctions, permeability, inflammation, and repair processes, and discuss the relevance of these changes to disease susceptibility, systemic inflammation, and climate-related health risks.

## Epithelial barrier theory

Epithelial barriers are the body’s first line of defense. They have diverse structures across organs but common features [50]. Cell-cell junctions strengthen the barrier, preventing paracellular leakage [51–53]. Chemical and immunological defenses are also part of these barriers. Epithelia cooperate with local immune cells, and a commensal microbiota resides on barrier surfaces [54, 55]. In healthy conditions, this system maintains tissue homeostasis and protects against allergens, microbes, and toxins. Epithelial barriers in the skin, gut, airways and nasal mucosa act as dynamic interfaces with the environment [56–59].

The epithelial barrier theory holds that the modern epidemic of chronic inflammatory diseases results from widespread disruption of the epithelial barrier due to environmental and lifestyle changes [34]. Proposed by Akdis, it explains the surge in allergic, autoimmune, and other chronic inflammations that take place in the initiation and exacerbation of many NCDs in the past 65 years [38]. Industrialization has introduced toxic substances and pollutants that damage epithelial barriers in the skin, respiratory tract, gut and other mucosal surfaces. Barrier defects and microbial dysbiosis trigger inappropriate immune activation, causing or worsening over 70 chronic inflammatory diseases [36, 60–62]. This theory builds on earlier studies, emphasizing, immune system interaction with the epithelial cells and environmental exposures [63–67]. Major environmental agents that compromise epithelial barrier integrity are given in Table 1. It also aligns with the One Health perspective as pets and livestock exposed to the same pollutants develop similar diseases [68, 69]. It links environmental changes to epithelial barrier injury and the rise of allergic and inflammatory diseases.

**Table 1:** Epithelial barrier disrupting substances and their effects

Category / Type of Agent	Examples	Major Effects on Health / Environment
Pathogens	Bacteria, fungi, virus, proteases, toxins	Respiratory diseases, infectious diseases, neurological diseases, and osteomyelitis, increase mortality risk [70].
Air pollutants	PM2.5, nitrogen dioxide, ozone, volatile organic compounds	Respiratory and cardiovascular diseases, oxidative stress, systemic inflammation, increased mortality risk [71, 72].
Household detergents and surfactants	Sodium lauryl sulfate, nonionic surfactants, cleaning agents, toothpastes	Skin and eye irritation, allergic responses, aquatic toxicity due to wastewater contamination [61, 73, 74].
Food additives *(emulsifiers*,* preservatives)*	Carboxymethylcellulose, polysorbates, sodium benzoate, food colorants, soy lecithin, DATEM	Altered gut microbiota, metabolic inflammation, potential links to obesity and inflammatory bowel disease [44, 75, 76].
Microplastics and nanoparticles	Polyethylene, polystyrene particles, titanium dioxide, nanoparticles	Cellular oxidative stress, inflammation, bioaccumulation in food chains, potential neurotoxicity [44].
Climate extremes and secondary pollutants	Wildfire smoke, heat waves, floods, humidity, ozone formation	Exacerbation of respiratory illness, heat-related mortality, contamination of water and food systems, displacement stress [77].

There is strong evidence linking epithelial barrier defects to the pathogenesis of many chronic diseases. Mechanistically, barrier damage starts an immune cascade. Stressed epithelial cells release alarmins and several chemokines and even activate the inflammasome that further activate several immune cells to accumulate in the periepithelial area, leading to chronic allergic inflammation, which further continues to impair the epithelial barrier [78–81]. Genetic mutations and environmental insults lead to a “leaky” epithelium, allowing allergens and microbes to penetrate and cause cutaneous Th2-driven inflammation [82]. AD shows how skin barrier dysfunction can cause inflammation [83]. A defective epithelial barrier is also observed in other allergic conditions such as asthma, allergic rhinitis, and chronic rhinosinusitis [71]. In asthma, the airway epithelium has impaired junctional integrity and heightened reactivity to pollutants and allergens, leading to chronic airway inflammation [84]. Nasal mucosal barrier disruption occurs in allergic rhinitis and chronic rhinosinusitis, often with microbial dysbiosis and type 2 inflammation [84, 85]. These diseases frequently coexist; for example, infants with eczematous skin damage are at higher risk of later food allergies and asthma [84, 86]. Epithelial leakiness causes allergen sensitization and the spread of inflammation [79, 81].

**Fig. 2:**
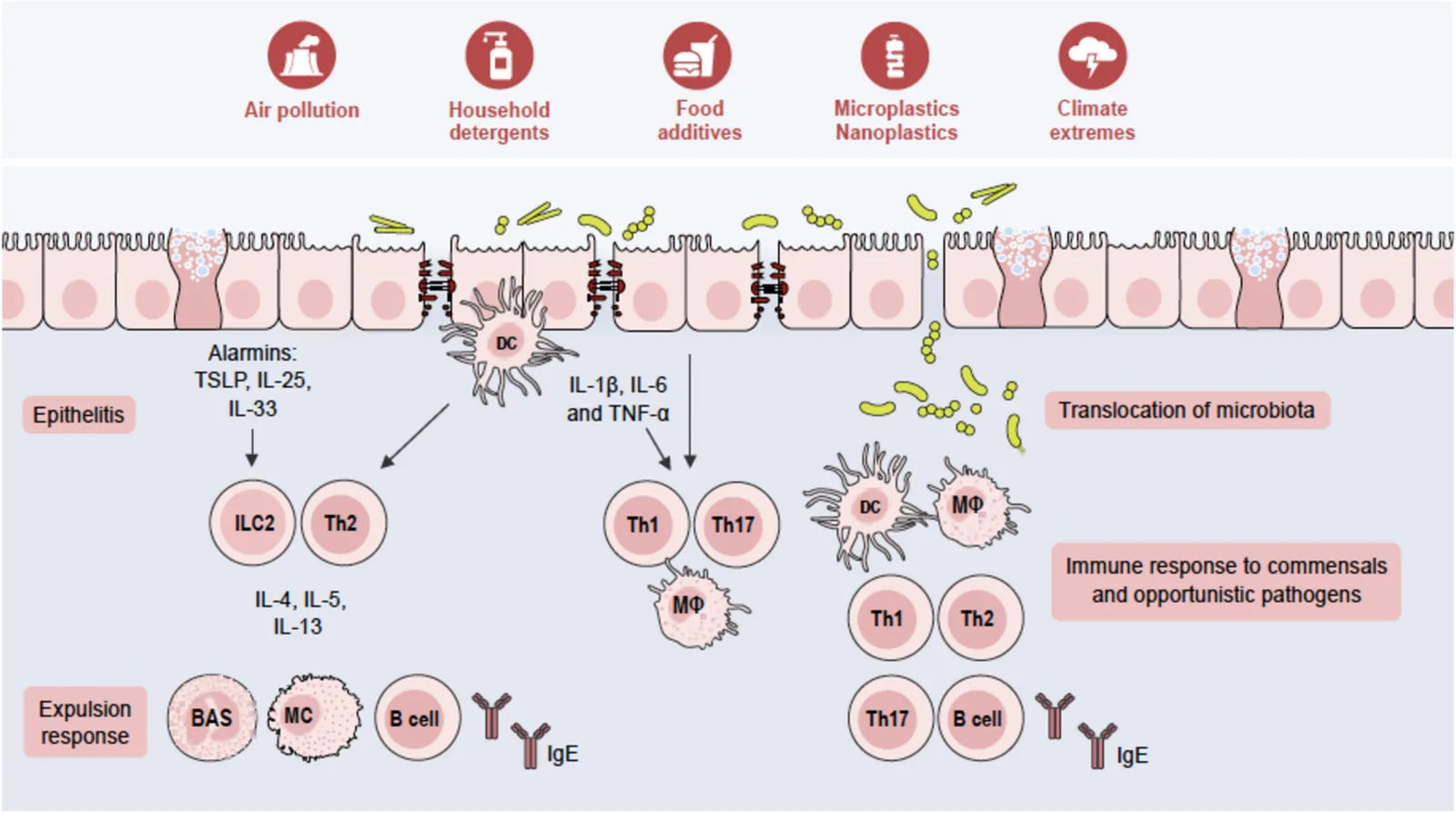
Environmental triggers and epithelial–immune interactions driving type 2 and mixed immune responses. The schematic overview illustrates how diverse environmental exposures—including air pollution, household detergents, food additives, microplastics, nanoplastics, and climate extremes—disrupt epithelial integrity and induce epithelitis. Damaged epithelial cells release alarmins (TSLP, IL-25, IL-33), which activate ILC2, DCs, macrophages (MΦ), basophils, and MCs. These pathways lead to downstream production of type 2 cytokines (IL-4, IL-5, IL-13), B-cell activation and IgE class switching, as well as Th2 differentiation. Concurrent activation of Th1 and Th17 pathways, driven by cytokines such as IL-1β, IL-6, and TNF-α, reflects a broader inflammatory response, including reactions to commensals, opportunistic pathogens, and microbiota translocation. Collectively, these processes contribute to expulsion responses and chronic airway inflammation. DC, dendritic cell; IL, interleukin; ILC, innate lymphoid cell; MC, mast cell; Th, T helper cell; TNF, tumor necrosis factor; TSLP, thymic stromal lymphopoietin

Beyond atopic disorders, barrier dysfunction is involved in many conditions. Upper and lower gastrointestinal barrier permeability is linked to food allergy, eosinophilic esophagitis, autoimmune and metabolic disorders [87, 88]. The neoexposome of the industrialized and modernized life challenges barrier resilience. Diseases under the epithelial barrier theory share features such as increased prevalence since the 1960s, epithelial defects and microbial imbalance, and chronic inflammation [58, 89, 90]. A second epidemic wave of food allergy and eosinophilic esophagitis started to develop after 2000s [91]. Several epithelial barrier diseases often appear as multimorbid clusters, indicating that barrier breakdown can cause diverse immunopathologies. To break the cycle of dysfunction and inflammation, limiting exposures and fortifying barriers have been recommended (Fig. 2). Protecting barrier interfaces appears to be crucial for preventing allergic and chronic inflammatory diseases in modern environments.

## Extreme temperatures as environmental stressors

### The effect of heat exposure to epithelial barriers

Extreme heat exerts direct biological effects on epithelial tissues, independent of secondary environmental exposures. Through temperature-driven molecular and cellular stress pathways, heat can compromise barrier function and heighten susceptibility to inflammation and allergic sensitization [92, 93]. Physiologically, elevated ambient temperatures lead to systemic dehydration and local drying of mucosal surfaces [94]. In the airways, this thermal stress impairs mucociliary clearance by altering the viscosity of the airway surface liquid, leading to the accumulation of particulate matter and pathogens [95]. In the skin, excessive sweating can lead to occlusion of ducts and maceration of the stratum corneum, physically weakening the barrier against external insults (96, 97).

Rising ambient temperatures are closely linked to increased ozone (O_3_) formation, which further exacerbates epithelial barrier damage. High temperatures can result in O_3_ pollution, leading to damage in the nasal epithelial barrier [98, 99]. Li et al. showed that the synergistic effect of O_3_ and high temperature would lead to exacerbate symptoms in allergic rhinitis mice [100]. Animal studies confirm that heat exposure rapidly compromises gut integrity, as evidenced by splanchnic endotoxemia in rats [101] and elevated endotoxin levels in primate plasma [102]. Plasma dextran assays in rodents further demonstrate heat-induced leakage [103–105].

### The effect of cold exposure to epithelial barriers

Conversely, cold environments also impose considerable stress on epithelial barriers. Epidemiological studies link low temperature to increased risks of respiratory diseases [106, 107]. A meta-analysis of 26 studies involving more than 26 million participants from 14 countries found that each 1 °C decrease in temperature was associated with a 5% higher risk of asthma exacerbation [106].

The mechanism of cold-induced injury is distinct from heat. Cold air is inherently dry, and its inhalation necessitates rapid humidification by the respiratory mucosa. This drives severe water loss from the airway surface liquid, causing local hypertonicity and osmotic shock to the epithelial cells [108]. Furthermore, cold exposure triggers localized vasoconstriction in the skin and nasal mucosa, reducing the delivery of leukocytes and plasma proteins necessary for barrier repair and immune defense [109]. Together, these results indicate that low temperature not only damages structural barrier proteins, but also modulates immune signaling, thereby amplifying inflammation in both respiratory and gastrointestinal systems.

### Synergistic effects with humidity, UV radiation and pollution

In real-life scenarios, epithelial barriers are rarely exposed to a single stressor. Rather, combined environmental factors, including air pollution, humidity, and ultraviolet (UV) radiation, interact to intensify tissue damage. Exposure to air pollution is responsible for an estimated 4.2 million deaths annually according to the World Health Organization (2024) [110]. It was shown that exposure to UV light, in combination with O_3_ and diesel exhaust emissions, synergistically enhances oxidative damage in human skin (111). This finding highlights the importance of combined exposures in evaluating environmental risk. Similarly, humidity significantly influences epithelial physiology. Both excessively low and high relative humidity can impair skin barrier function by altering filaggrin metabolism, increasing protein deamination, and weakening the stratum corneum [112].

Occupational settings are an important real-world example of compound barrier injury because many workers experience extreme temperatures together with chemical, particulate, biological and physical stressors during long exposure windows and with limited opportunity for recovery. Agricultural workers may experience high radiant heat, dehydration, dust, pollen, soil-derived microbial products and pesticide exposure during prolonged outdoor labor, creating simultaneous thermal, chemical and particulate stress on the skin, airway and intestinal barriers. Heat increases sweating and salt loss, while dust and agrochemicals can irritate the respiratory mucosa and skin surface; together, these exposures may weaken tight junctions, alter mucus properties, increase oxidative stress and promote epithelial alarm signaling. Firefighters represent an acute high-intensity exposure group. They face extreme heat together with wildfire smoke rich in PM2.5, PAHs, VOCs, metal-containing particles and combustion-derived irritants, which can damage the respiratory epithelium while sweating, friction, occlusive protective equipment and repeated wet-dry cycles weaken the cutaneous barrier. Factory, foundry, construction, mining, warehouse and other industrial workers may experience high indoor or outdoor heat in combination with solvents, metal fumes, welding emissions, combustion products, cement dust, cleaning chemicals or microplastic-containing aerosols. In these environments, the barrier-relevant insult is not simply heat load; it is the repeated overlap of dehydration, local epithelial drying, inhaled irritants, dermal contamination and systemic inflammatory strain. Occupational hazards severely compromise the skin barrier’s stratum corneum, leading to occupational skin diseases [113, 114]. Extreme temperatures structurally alter this protective shield; cold environments cause micro-cracking and trigger inflammatory cytokine release, while extreme heat and sweat macerate tissue and disrupt essential lipids [115, 116]. Concurrently, workplace chemical irritants dissolve the intercellular lipid matrix, accelerating the dermal absorption of toxins. Pollutants like PM2.5 deplete crucial filaggrin proteins, inducing profound oxidative stress and driving barrier failure [10].

Occupational heat stress also promotes renal strain, reduced plasma volume and cardiovascular load, indicating that epithelial injury may occur alongside broader systemic vulnerability in exposed workers [117]. This is particularly important because workers with asthma, allergic rhinitis, atopic dermatitis, inflammatory bowel disease or cardiometabolic disease may have reduced reserve to tolerate repeated epithelial stress. Protective equipment can reduce inhalational and dermal exposures but may also increase heat retention, sweat accumulation and skin maceration if not paired with cooling strategies, rest periods and hydration. The occupational context therefore provides a concrete model of multi-hit barrier damage: thermal stress increases epithelial susceptibility, pollutants or toxins provide a direct second insult, and repeated work cycles may prevent complete repair. Recognizing these interactions can guide prevention through exposure monitoring, heat-action thresholds, ventilation, task rotation, respirator selection, skin protection, hydration protocols and post-exposure medical surveillance.

Collectively, these studies emphasize that the epithelial barrier acts as a critical interface between the body and the external environment. Extreme temperatures, whether heat or cold, compromise epithelial integrity through oxidative stress, inflammation, and altered protein regulation. Moreover, co-exposure to other environmental stressors, such as UV radiation, humidity fluctuations, and air pollution, can amplify these deleterious effects. Understanding these synergistic mechanisms is essential for predicting health outcomes under changing climate conditions and for developing targeted interventions to preserve epithelial barrier homeostasis.

## Pollution, environmental stressors, and the shifting exposome

**Fig. 3:**
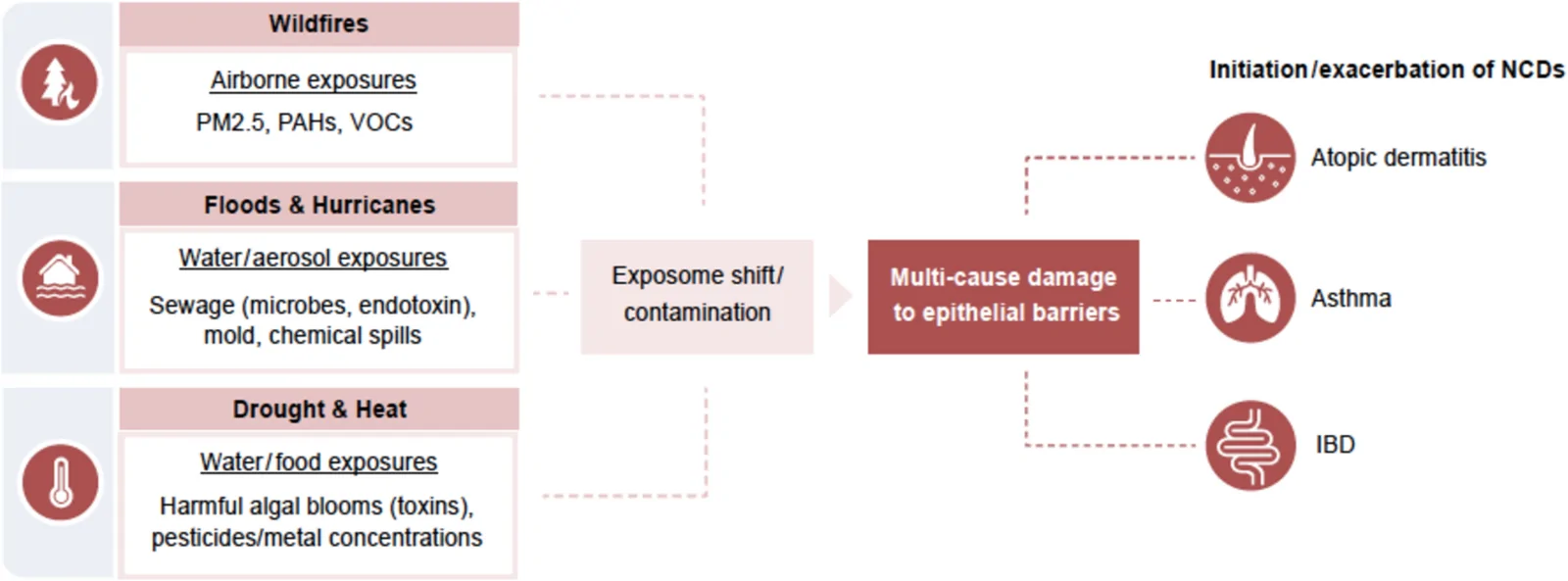
Altered weather–associated environmental events shift the exposome and drive “multi-hit” epithelial barrier damage. Major climate events create distinct, complex pollutant mixtures that breach specific barriers. Wildfires generate airborne plumes of PM2.5, PAHs, and VOCs that compromise respiratory and skin barriers. Floods and hurricanes create toxic waters and aerosols containing sewage-derived microbes and endotoxins, mold, and chemical spills, challenging lung and skin integrity. Drought and extreme heat concentrate contaminants in water and food systems, including toxins from harmful algal blooms and heavy metals or pesticides, which directly damage the intestinal barrier. These combined exposures result in a “multi-hit” assault on epithelial tissues, leading to the initiation or exacerbation of NCDs such as AD, asthma, and IBD. AD, atopic dermatitis; IBD, inflammatory bowel disease; NCDs, noncommunicable diseases; PAHs, polycyclic aromatic hydrocarbons; PM2.5, particulate matter ≤ 2.5 μm; VOCs, volatile organic compounds

The health impacts of temperature extremes and altered weather patterns cannot be understood by studying single stressors in isolation. These changes are fundamentally and rapidly altering the exposome, the totality of all environmental exposures an individual encounters from conception to death [118]. This shifting exposome is characterized not only by the intensification of known stressors, such as heat, but also more critically, by novel and complex mixtures of exposures arising from climate-driven environmental catastrophes. Events such as wildfires, floods, and droughts and extreme precipitation. These events act as exposure amplifiers, creating hazardous combinations of physical, chemical, and biological agents that deliver a multi-pronged assault on epithelial barriers (Fig. 3; Table 2).

**Table 2:** The mechanisms of the shifting exposome

Climate Change Driver	Resulting Environmental Event	Contamination/Shifting Exposome	Affected Barrier(s)	Barrier Change /Dysfunction
Hotter, Drier Conditions / Extreme heat	Wildfires, heatwaves and occupational or outdoor heat exposure	Complex mixture of PM2.5, PAHs (polycyclic aromatic hydrocarbons), VOCs, and heavy metals, concentrated soil/water contaminants	Respiratory (Lung); Intestinal (Gut); Cutaneous (Skin)	Severe oxidative stress; disruption of tight junctions (TJs); impaired mucociliary clearance; direct pathway for toxicant/microbe breach, intestinal splanchnic vasoconstriction, ischemia, tight-junction loss and LPS translocation; skin sweating, maceration and barrier weakening.
Extreme Precipitation	Floods	Toxic mixture (aerosolized): Microbes (sewage: E. coli, Vibrio), chemical runoff (pesticides, solvents), fungal spores (mold), microplastics	Respiratory (Lung), Cutaneous (Skin)	Breach by microbial/chemical agents; drives acute infections and chronic inflammatory sensitization.
Extreme Precipitation	Water Supply Contamination	Waterborne gut pathogens (e.g., Cryptosporidium, Giardia, pathogenic E. coli) overwhelm treatment facilities.	Intestinal (Gut)	Direct pathogen challenge to the intestinal barrier; alteration of the gut microbiome.
Rising Water Temperatures	Harmful Algal Blooms (HABs)	Potent toxins (e.g., microcystins, ciguatoxins) accumulate in drinking water and seafood.	Intestinal (Gut)	Directly cytotoxic (damaging) to the gut epithelial cells; promotes liver/neurotoxicity.
Droughts	Concentration of Contaminants	Heavy metals and pesticides become concentrated in remaining soil and surface water.	Intestinal (Gut)	Chronic, low-dose ingestion via the food chain, contributing to gut barrier dysfunction.
Extreme Weather Events	“Thunderstorm Asthma”	Osmotic shock ruptures pollen grains, creating a high-concentration aerosol of respirable-sized allergens.	Respiratory (Lung)	Overwhelms the barrier with a massive, sudden allergen load, triggering widespread immune activation.

Wildfires, which are increasing in frequency and scale due to hotter and drier conditions, are a primary example. Wildfire smoke is not equivalent to typical urban air pollution. It is a highly complex and dynamic mixture of fine particulate matter (PM2.5), carbon monoxide, polycyclic aromatic hydrocarbons (PAHs), volatile organic compounds (VOCs), and heavy metals, generated from the combustion of both biomass and, increasingly, man-made structures (the wildland-urban interface) [119]. This unique composition is exceptionally toxic to the respiratory epithelium. Many of these chemicals, particularly PAHs, are potent ligands for the Aryl hydrocarbon receptor (AhR). Upon binding, the AhR-ligand complex translocates to the nucleus, driving the expression of metabolic enzymes (CYP1A1, etc.) that, while attempting to detoxify the pollutants, generate a massive burst of reactive oxygen species (ROS) [120]. In both mouse models and human lung epithelial cells, this oxidative stress, combined with direct AhR signaling, activates pro-inflammatory pathways such as NF-κB and MAPK, which, in turn, promote the phosphorylation and delocalization of tight junction proteins such as ZO-1 and occludin [121]. This molecular cascade disrupts tight junctions, paralyzes mucociliary clearance, and provides a direct paracellular pathway for toxicants and co-inhaled microbes to breach the barrier [119, 121, 122]. With widespread epithelial barrier injury, wildfire exposures act as a systemic exposome stressor, contributing to diseases across multiple organ systems (including respiratory, cardiovascular, and skin) [35, 123].

Beyond standard combustion products, wildfire smoke acts as a carrier for toxic metals and per- and polyfluoroalkyl substances (PFAS), significantly altering the immunologic landscape [34]. Our recent study revealed a strong association between smoke exposure and differential methylation at 133 disease-relevant gene loci. Immune phenotyping further demonstrated increased expression of immune cell homing and activation markers. We also developed a mass cytometry–based approach to quantify metal accumulation at the single-cell level and observed elevated mercury concentrations in dead cells and increased cadmium levels in both viable and nonviable cell populations. Interestingly, mercury accumulation correlated with the duration of smoke exposure. In addition, multiple epigenetic loci across different chromosomes were significantly associated with specific toxic metal isotopes within individual immune cells [124]. Together, these single-cell–level methods and findings provide mechanistic insight into the biological impact of smoke exposure and may facilitate the identification of exposure timing and molecular targets that could be leveraged for prevention and therapeutic intervention.

Climate-driven floods and extreme precipitation events present a different, but equally hazardous, exposure profile. Floodwaters are a toxic mixture that aerosolizes a myriad of barrier-disrupting agents. This includes microbial contaminants from overwhelmed sewage systems (*E. coli*, *Vibrio* species, endotoxin, etc.), chemical runoff from agricultural and industrial sites (pesticides, herbicides, industrial solvents), and, critically, fungal spores from water-damaged buildings (*Aspergillus*, *Stachybotrys*, etc.) [125]. Inhalation or contact with these aerosols and contaminated water poses a severe threat to the lung and skin barriers, driving both acute infections and chronic inflammatory sensitization.

These exposome shifts also contaminate the foundational elements of public health: water and food. Floods routinely overwhelm water treatment facilities, leading to widespread contamination of drinking water with gut pathogens (such as *Cryptosporidium*, *Giardia*, pathogenic *E. coli*) that directly challenge the intestinal barrier and alter the microbiome [126]. In parallel, rising water temperatures promote the proliferation of harmful algal blooms (HABs) in both freshwater and marine environments. These blooms produce potent toxins, including microcystins in lakes and ciguatoxins in oceans, that accumulate in drinking water and seafood [127]. When ingested, these toxins directly damage the gut epithelium and can promote liver damage and neurotoxicity. Similarly, droughts can concentrate heavy metals and pesticides in soils and surface water. Droughts also intensify the frequency of dust storms, which transport mineral dust, endotoxin-containing bioaerosols, fungal spores, and allergens over vast distances [118, 128, 129]. These particles can mechanically irritate the airway surfaces, expose epithelial cells to microbial ligands and allergenic proteins and amplify innate and type 2 immune signaling [130]. This accumulation of contaminants in the food chain and air results in chronic, low-dose exposure that can contribute to gut and lung barrier dysfunction [131].

This climate change-driven “multi-hit” model provides a mechanistic link to the rising prevalence of NCDs. The epithelial barrier is simultaneously weakened by a physical stressor such as heat or cold and further damaged by a novel chemical/biological stressor like wildfire-derived PAHs or floodwater-derived mold. This synergistic assault can overwhelm the barrier’s homeostatic repair mechanisms, initiating a cycle of chronic inflammation. This framework is supported by recent epidemiological studies. Exposure to wildfire smoke is consistently associated with sharp increases in emergency department visits and hospitalizations for respiratory diseases, including asthma and chronic obstructive pulmonary disease exacerbations [132]. The systemic impact is also clear, with studies linking wildfire PM2.5 exposure to an increased immediate risk of out-of-hospital cardiac arrests and other acute cardiovascular events [133]. Furthermore, emerging data show a significant association between wildfire smoke exposure and increased outpatient visits for inflammatory skin conditions, particularly atopic dermatitis, suggesting a breach of the cutaneous barrier [14]. This framework helps explain epidemiological observations, such as the rise in “wildfire asthma” or “thunderstorm asthma,” in which pollen grains are ruptured by osmotic shock during storms, releasing a potent aerosol of respirable-sized allergens that triggers widespread exacerbations [134, 135]. It also explains post-hurricane spikes in eczematous skin conditions and gastrointestinal distress. The shifting exposome, therefore, acts as the critical bridge connecting large-scale environmental events to the individual-level immunopathology of NCDs [136].

The mechanisms of the shifting exposome are shown, linking primary climate change drivers to specific environmental events. These events generate novel mixtures of contaminants that directly assault and compromise the body’s epithelial barriers (lung, gut, and skin), leading to specific types of dysfunction. Abbreviations: PAHs, polycyclic aromatic hydrocarbons; PM2.5, particulate matter ≤ 2.5 μm); TJs, tight junctions; VOCs, volatile organic compounds.

## Mechanistic pathways linking temperature and epithelial barrier dysfunction

### Overview of temperature-barrier interactions

**Fig. 4:**
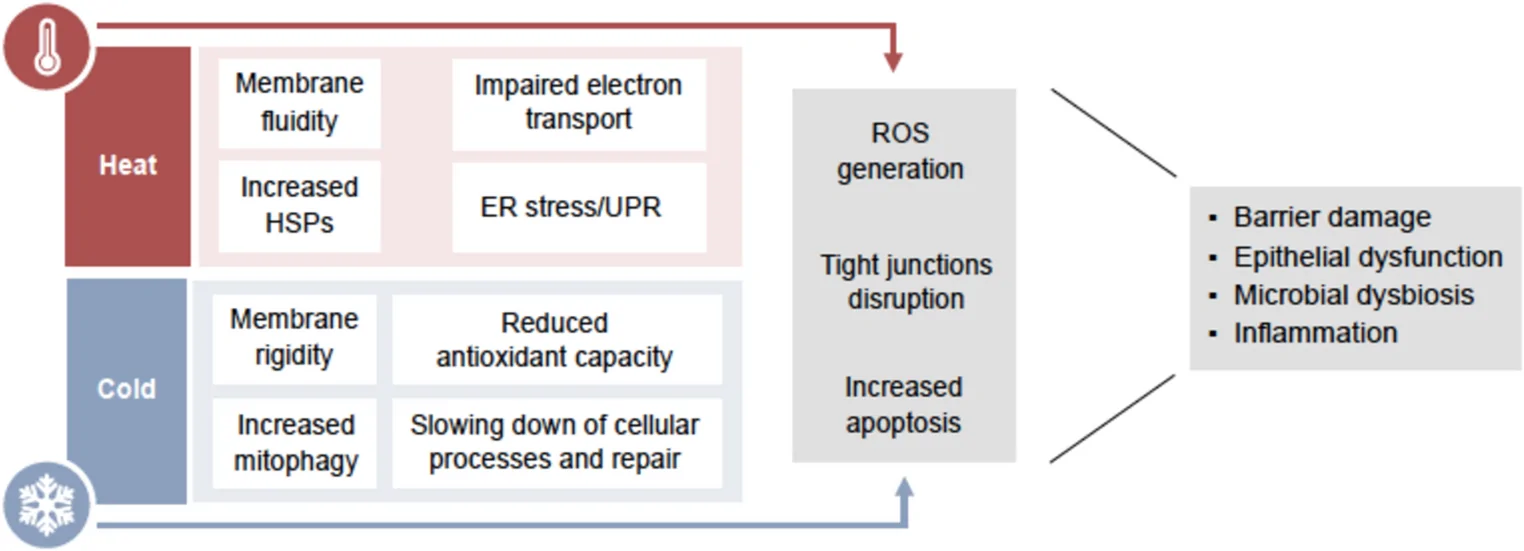
Schematic overview of temperature-induced epithelial barrier dysfunction. Heat stress promotes ROS generation, mitochondrial electron transport impairment, an increase in ER stress/UPR, tight junction disruption, altered membrane fluidity, and apoptosis, whereas cold stress slows cellular processes and repair, increases mitophagy, reduces antioxidant capacity, and rigidifies membranes. Together, these thermal stress pathways converge on barrier damage, epithelial dysfunction, microbial dysbiosis, and inflammation. ER, endoplasmic reticulum; ROS, reactive oxygen species; UPR, unfolded protein response

Extreme temperatures trigger complex stress responses at the cellular level, activating molecular, biochemical, and immunological pathways that protect against damage to proteins, nucleic acids, and membranes. Both heat and cold disrupt redox balance, energy metabolism, and membrane fluidity, which weakens epithelial stability. Heat stress causes protein denaturation and loss of enzymatic activity, triggering the heat shock response [17, 137]. In contrast, cold impairs protein folding and slows metabolic processes; cells respond by upregulating cold shock proteins to preserve RNA integrity and cytoskeletal [138, 139]. Temperature extremes also alter membrane dynamics; heat increases lipid fluidity and permeability, while cold rigidifies membranes and restricting transport [140]. Both forms of stress enhance oxidative imbalance: heat accelerates mitochondrial respiration and ROS production, whereas cold reduces antioxidant capacity and repair efficiency [141, 142]. Meanwhile, thermal extremes affect immune activity through inflammatory signaling and stress-related cytokine release. While heat can activate innate and adaptive immune pathways, evidence in teleost models show that it may also suppress immune cell function [143]. Together, oxidative injury, mitochondrial failure, and immune dysregulation converge to disrupt epithelial homeostasis (Fig. 4). This integrated framework explains temperature-induced barrier breakdown observed in multiple organs.

### Cellular stress responses to temperature changes

**Fig. 5:**
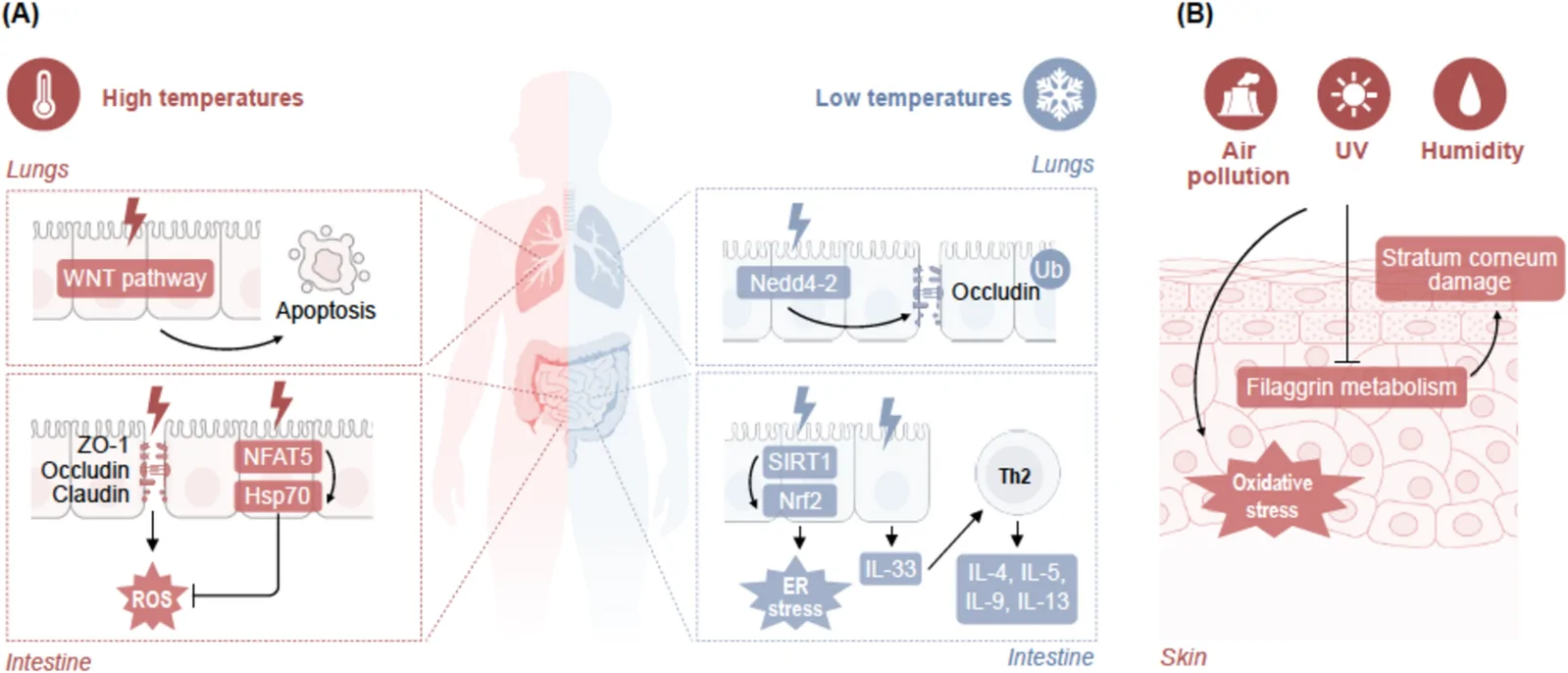
Both high and low temperatures can impair the epithelium through the activation of various cellular pathways. (A) Impact of extreme temperatures on lung and intestinal barriers. High temperatures induce apoptosis in lung epithelial cells via the Wnt signaling pathway. In the intestine, heat stress disrupts tight junction proteins (ZO-1, occludin, claudin) and increases reactive oxygen species (ROS); conversely, the NFAT5–Hsp70 pathway is activated to mitigate ROS and support regeneration. Low temperatures compromise lung barrier integrity by promoting Nedd4-2-mediated ubiquitination and degradation of occludin. In the intestine, cold stress induces endoplasmic reticulum (ER) stress via the SIRT1/Nrf2 pathway and drives type 2 inflammation (IL-4, IL-5, IL-9, IL-13) through IL-33-mediated Th2 cell activation. (B) Synergistic effects of environmental co-exposures on the skin. Air pollution directly triggers oxidative stress, while UV radiation interferes with filaggrin metabolism. Humidity fluctuations cause physical damage to the stratum corneum, further disrupting filaggrin processing and amplifying oxidative injury. ER, endoplasmic reticulum; Hsp, heat shock protein; IL, interleukin; Nedd4-2, neural precursor cell expressed developmentally down-regulated protein 4 − 2; NFAT, nuclear factor of activated T cells; Nrf2, nuclear factor erythroid 2–related factor; ROS, reactive oxygen species; SIRT, sirtuin; Th, T helper cell; Ub, ubiquitin; UV, ultraviolet; Wnt, Wingless-related integration site; ZO-1, zonula occludens-1

Epithelial cells sense and respond to thermal stress through interconnected pathways involving oxidative stress, mitochondrial dysfunction, ER stress, and the unfolded protein response and multiple cell death mechanisms. Hyperthermia can induce respiratory epithelial apoptosis and stimulate epithelial-to-mesenchymal transition (EMT)-related gene expression in human lung epithelium by the Wnt pathway [144, 145]. These processes collectively cause barrier dysfunction in the skin, airway, and gut epithelia (Fig. 5).

Thermal damage to proteins and nucleic acids activates HSPs, which help refold denatured proteins and maintain cell integrity. Interestingly, nuclear factor of activated T cells (NFAT) 5 enhances intestinal epithelial regeneration by inducing HSP70, protecting cells from heat‑induced and inflammatory injury [146]. However, when overwhelmed, the accumulation of HSPs at the cell membrane initiates a pro-inflammatory cascade. These events include oxidative stress, increased NF-kB expression, unfolded protein response, setting the cell prone to inflammasome activation and dysregulated cell death.

Both heat and cold increase ROS production, leading to oxidative damage to DNA, proteins, and lipids [147–151]. ROS-mediated oxidative damage can trigger apoptosis if unrestrained [152]. Heat disrupts mitochondrial respiration by suppressing OXPHOS and electron transport chain activity, increasing electron leak and ROS accumulation [153–155]. Cold activates mitophagy and HIF-1α signaling as compensatory responses [156]. Heat-induced ROS also elevates cytosolic calcium, activating Bcl-2–dependent apoptotic cascades and Bax translocation [157–160], while the RIPK1/RIPK3/MLKL axis drives necroptosis in severe heat stress [159, 161]. Cold exposure induces apoptosis and can trigger ferroptosis through ASK1–p38 MAPK signaling [162].

Human studies similarly show heat-induced upregulation of *HSPA1A* (encoding HSP70-1) and activation of the unfolded protein response, key mechanisms conferring thermotolerance [163, 164]. In the intestinal epithelial barrier, cold exposure leads to barrier damage and endoplasmic reticulum (ER) stress by inducing the SIRT1/Nrf2 signaling pathway [165]. Together, these processes contribute to epithelial cell loss, mitochondrial collapse, and chronic tissue inflammation. For example, heat shock reduces E-cadherin expression and induces partial EMT [166].

### Molecular regulation of barrier function due to temperature changes

Heat stress markedly compromises epithelial integrity by disrupting tight junction complexes. High temperatures reduce transepithelial electrical resistance and downregulate key junctional proteins including ZO-1, occludin, and Claudin-3 [167, 168]. Concurrently, MAPK activation and oxidative stress amplify junctional disorganization [168–170]. Heat shock proteins (HSPs), particularly HSP70, play a protective role by maintaining protein homeostasis and stabilizing junctional complexes.

Cold stress, though less studied, disrupts membrane fluidity, metabolism, and protein function, indirectly destabilizing tight and adherens junctions [171]. Specifically, Zhou et al. found that cold exposure impairs bronchial epithelial integrity by inducing occludin degradation via a Nedd4‑2-dependent proteolytic mechanism [172]. Additionally, both heat and cold alter actin and microtubule architecture through Rho-GTPase–mediated remodeling, impairing cell polarity and mechanical cohesion [173–175].

Thermosensitive transient receptor potential (TRP) channels also participate in barrier regulation. TRPV1 and TRPV4 upregulation under high temperature correlates with epithelial permeability changes [176, 177]. Crucially, this activation exacerbates allergic inflammation; exposure to 35 °C upregulates TRPV1, leading to elevated serum IL-4, IL-6, TNF-α, and IgE, shifting the immune profile toward a Th2 response [178, 179]. In the skin, heat-activated TRPV3 triggers the secretion of thymic stromal lymphopoietin (TSLP) nerve growth factor and prostaglandin E_2_, driving the itch-scratch cycle and barrier breakdown in atopic dermatitis [180].

Overall, thermal extremes disrupt the molecular scaffolding that maintains epithelial cohesion. Junctional remodeling, cytoskeletal reorganization, and TRP signaling converge to destabilize the structure and increase permeability, key steps connecting temperature stress to inflammation.

### Endocrine responses to temperature changes and their effects on barriers

Temperature changes not only directly influence the epithelium but also lead to adaptations in the organism that may affect barrier integrity. The major adaptations occur through the endocrine system. Increases in core body temperature lead to the release of growth hormone [181], prolactin [182] and activate the hypothalamic-pituitary-adrenal (HPA) axis and the sympathetic nervous system [183]. This results in the secretion of adrenocorticotropic hormone, cortisol and norepinephrine. With increasing body temperature, activation of the renin-angiotensin-aldosterone system can also be observed [184]. In hypothermia, the significant adaptive responses are an increase in the production of thyroid hormones and activation of the HPA axis.

Although studies are conflicting on the effects of glucocorticoids on the barrier, chronic exposure to elevated cortisol levels impairs the barrier by downregulating of tight junction proteins. Exogenous administration of glucocorticoids in the presence of inflammation increases barrier integrity and downregulates inflammation [185]. Effectors of the stress response (cortisol, norepinephrine) lead to increased barrier integrity in the acute phase, while with prolonged, chronic exposures, result in impaired epithelial healing, loss of tight junction integrity, mitochondrial dysfunction, and microbial dysbiosis [186, 187].

Growth hormone has been shown to protect the intestinal barrier by increasing tight junction proteins, reducing permeability and bacterial translocation. Some studies have indicated that growth hormone treatment can mitigate intestinal damage caused by trauma, sepsis and surgery [188, 189].

The renin-angiotensin-aldosterone system (RAAS) significantly impacts the integrity of the intestinal barrier. Activation of the AT1 receptor by angiotensin II is associated with the release of inflammatory mediators, apoptosis and intestinal mucosal dysfunction. Whereas activation of the AT2R has anti-inflammatory properties [190, 191]. RAAS dysregulation, as observed in pathologies such as inflammatory bowel disease, leads to increased local aldosterone levels and altered electrolyte transport, resulting in exacerbated inflammation and barrier damage [192].

Thyroid hormones play a crucial role in regulating the intestinal barrier. In Graves’ disease, which is associated with hyperthyroidism, increased levels of circulating biomarkers related to gut leakiness have been identified [193]. Hypothyroidism has also been linked with decreased barrier integrity both in clinical and pre-clinical studies. Reduced thyroid hormones compromise intestinal barrier integrity and alter microbial composition, leading to increased bacterial translocation. One possible contributor to this effect is the thyroid hormones’ effect on gut motility [194, 195].

Temperature-induced endocrine adaptations significantly influence epithelial barrier integrity. While acute hormonal responses, such as transient stress reactions, may initially strengthen the barrier as a protective measure, chronic activation often disrupts tight junctions, impairs healing and triggers inflammation. Understanding how these systemic responses to thermal stress affect epithelial homeostasis is essential for uncovering the complex relationship between environmental temperature changes, endocrine regulation, and barrier-related diseases.

### Immune–epithelial interactions affected by temperature changes

Heat stress, whether from exertion or direct exposure, elevates circulating IL-6 and TNF-α [196]. In keratinocytes, hyperthermia induces transcription of TNF-α, IL-1β, TNFAIP3, and IL-8 [197]. Cold atmospheric plasma similarly increases IL-6, IL-8, TNF-α, VEGF, and interferon-γ expression, accompanied by mitochondrial dysfunction and lysosomal leakage [198]. In most epithelial tissues, IL-1β, IL-6, and TNF-α are rapidly induced by MAPK and NF-κB signaling in response to both heat and cold, modulating immune activation and barrier permeability [199].

Thermal stress also alters innate immunity. Heat enhances TLR2 and TLR4 expression in monocytes and peripheral blood cells, amplifying responsiveness to PAMPs and DAMPs [200, 201]. While extremely high temperatures can impair neutrophil accumulation and functional capacity [202] exposure to fever-range hyperthermia augments neutrophil recruitment to the lungs through HSP-regulated CXCL8 (IL-8) expression under HSF1 control, potentially exacerbating local inflammation and tissue injury (4, 203, 204, 205).

Likewise, heat exposure upregulates TLR2/4 and MHC molecules in dendritic cells, along with co-stimulatory receptors CD80 and CD86, driving Th1-polarizing cytokines IL-12 and TNF [206–210]. On the other hand, cold exposure exerts suppressive effects on immune activity. Rats exposed to 4 °C–− 12 °C show reduced IFN-γ, IL-2, IL-4, and TLR4 expression, along with fewer CD4⁺CD25⁺Foxp3⁺ Tregs [211]. Sub-optimal temperature also increases airway hyperreactivity and Th2 polarization of CD4⁺ T cells via upregulation of IL-4, IL-1β, IL-6, and TNF-α [212]. Moreover, cold stress upregulates IL‑33 expression in intestinal epithelial cells, promoting Th2 polarization and subsequent upregulation of IL-4, IL-5, IL-9 and IL-13 ultimately increasing allergic sensitization [213].

Both heat and cold thus drive profound dysregulation of innate and adaptive immune responses, leading to secondary impairment of epithelial barrier integrity in the skin, airways, and gut. At the same time, direct thermal injury independently compromises the barrier structure itself. In bovine intestine, heat stress disrupts intestinal epithelial tight junctions, increases permeability, facilitating microbial translocation that provokes macrophage activation and pro-inflammatory cytokine release [214]. In mice, while intragastric cold exposure induces colitis-like phenotypes, elevates gut permeability and reduces bacterial diversity [215].

Collectively, these findings highlight that extreme temperatures perturb epithelial integrity through overlapping oxidative, mitochondrial, and immune pathways. Thermal stress elicits cytokine storms, TLR activation, and immune cell infiltration while simultaneously weakening junctional complexes and organelle function. This interplay establishes a self-reinforcing cycle of inflammation and epithelial injury, a central mechanism linking environmental temperature extremes to chronic inflammatory, allergic, and metabolic diseases. Despite strong experimental and animal evidence, data in humans remain limited, with most insights derived from in vitro or short-term exposure studies. Expanding human-based multi-omic and organ-on-chip models will be crucial to understanding how chronic temperature fluctuations contribute to epithelial barrier dysfunction and NCD risk in real-world settings.

## Clinical and epidemiological evidence and implications for human health

The mechanistic pathways detailed above provide a strong biological basis for the “temperature-barrier-disease” axis. This plausibility is substantiated by a large and growing body of clinical and epidemiological research linking real-world temperature extremes to NCDs (Table 3). This evidence demonstrates that thermal stress is not merely a theoretical risk but a tangible, population-level driver of chronic disease morbidity and exacerbation.

**Table 3:** Summary of epidemiological and clinical evidence linking thermal stress to NCDs

Stressor	Barrier	Epidemiological / Clinical Evidence	Associated NCDs	Proposed Mechanism
Extreme heat	Pulmonary	↑Hospital admissions & ED visits for asthma/COPD during heatwaves. Synergistic effect with ozone & pollen.	Asthma, COPD	Dehydration of airway mucus. ↑Co-exposure to ozone (barrier toxicant).
	Intestinal	↑Hospitalization rates for IBD flares post-heatwave. ↑Incidence of infectious gastroenteritis.	IBD, Infectious Enteritis	Splanchnic vasoconstriction →Ischemia →↑Permeability (LPS translocation). ↑Pathogen replication.
	Cutaneous	↑ED visits for atopic dermatitis, miliaria. Exacerbation of inflammatory skin diseases.	Atopic Dermatitis, Miliaria	Sweat duct occlusion. Skin maceration & impaired barrier function.
Extreme Cold	Pulmonary	↑Incidence of viral respiratory infections (flu, rhinovirus). ↑Hospital admissions for asthma/COPD.	Asthma, COPD, Viral Infections	↓Mucociliary clearance. ↓Innate antiviral immunity in nasal passages. Cold-induced osmotic stress →mediator release & bronchoconstriction.
	Cutaneous	Clear seasonal pattern of AD & psoriasis flares (winter). “Winter Xerosis” (dry skin).	Atopic Dermatitis, Psoriasis	↑Transepidermal Water Loss (TEWL). ↓Ceramide/lipid synthesis due to vasoconstriction.
Wildfires, floods, droughts and thunderstorm-related allergen events	Pulmonary, cutaneous and intestinal barriers	Increased asthma events after wildfire smoke exposure; skin and gut infections after flooding, allergen surges during thunderstorm asthma; dust-related fungal and endotoxin exposure	Asthma, allergic sensitization, infectious enteritis, inflammatory skin disease	Specific multi-hit exposures; PM2.5 plus heat affect lung and skin; sewage, endotoxin and mold after floods affect lung, skin and gut; dust storms transport fungal spores, endotoxin and respirable allergen fragments.

### Extreme heat exposure and increased risk of multisystem disease exacerbations

Robust data linking temperature and NCDs often come from time-series analyses of hospital admissions and emergency department visits during and after periods of extreme heat [216, 217]. These acute, high-impact heat events are usually studied as natural experiments that reveal the immediate health consequences of thermal stress, particularly in vulnerable populations [218].

**Fig. 6:**
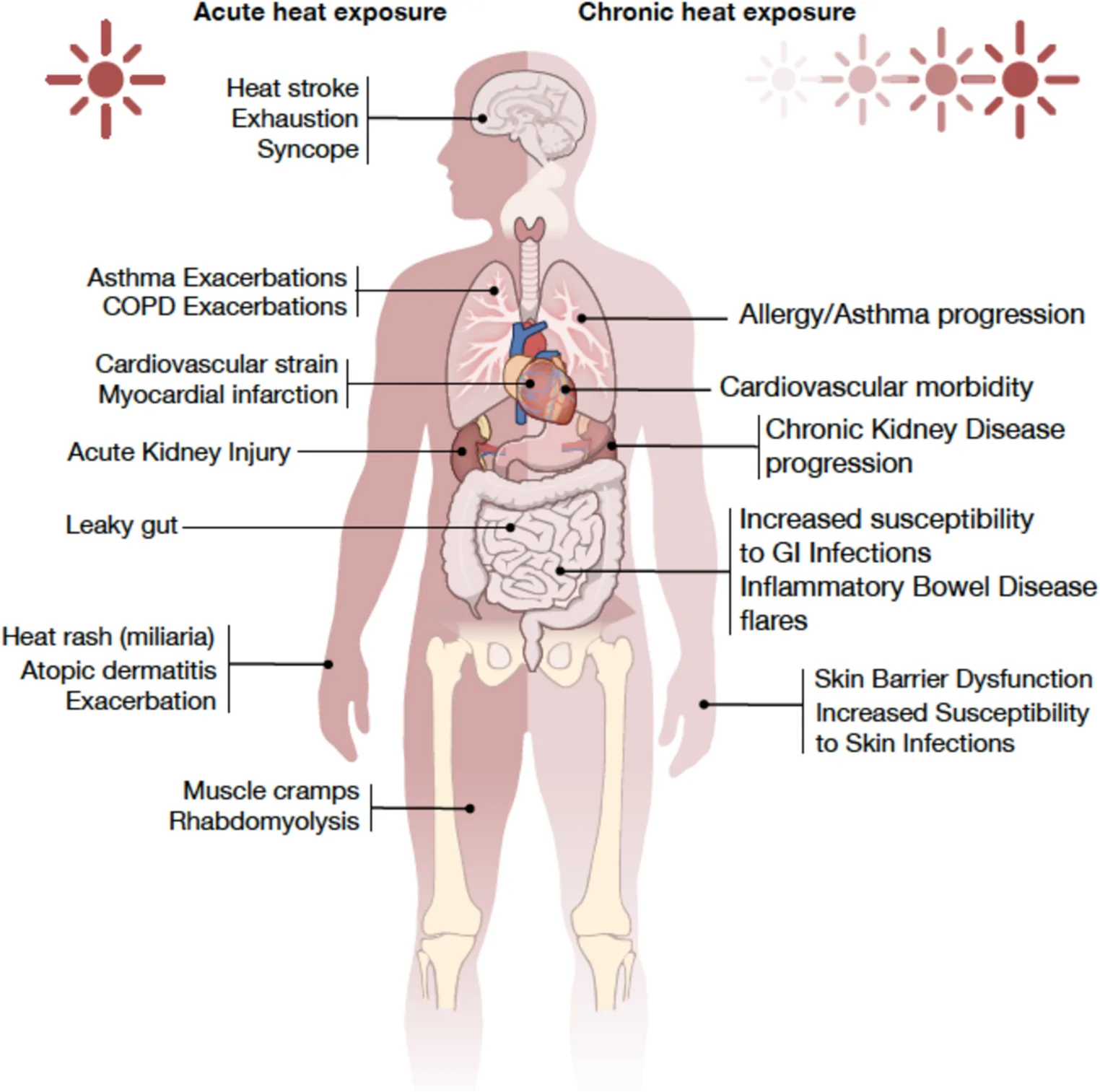
The systemic health impacts of heat exposure, stratified by exposure duration. The figure is divided vertically to contrast the health impacts of short-term versus long-term heat stress. The left panel (acute heat exposure) details immediate physiological complications, including neurological impacts (heat stroke, exhaustion, syncope), respiratory exacerbations (asthma, COPD), cardiovascular strain and myocardial infarction, acute kidney injury, gastrointestinal permeabilization, dermatological issues (heat rash, atopic dermatitis exacerbation), and musculoskeletal damage (muscle cramps, rhabdomyolysis). The right panel (chronic heat exposure) outlines long-term disease progression and increased susceptibility. Key effects include allergy and asthma progression, cardiovascular morbidity, chronic kidney disease progression, gastrointestinal vulnerabilities (increased susceptibility to GI infections, inflammatory bowel disease flares), and skin barrier dysfunction with a higher risk of skin infections. COPD, chronic obstructive pulmonary disease; GI, gastrointestinal

Multiple large-scale cohort and case-crossover studies have identified a consistent, positive association between high ambient temperatures and exacerbations of both asthma and COPD (Fig. 6). A nationwide case-crossover study in England found that for every 1 °C increase in summer temperatures above 23.2 °C, the risk of COPD hospitalization increased by 1.47% [219]. The mechanism is often synergistic, as heatwaves accelerate photochemical reactions that produce ground-level ozone [117], a respiratory irritant that induces airway inflammation and disrupts epithelial tight junctions [220]. Furthermore, high heat can increase pollen counts and the aerosolization of fungal spores, creating a “multi-hit” exposure of thermal stress, chemical pollutants, and biological allergens that collectively overwhelm the pulmonary barrier [221, 222].

The link between heat and intestinal disease is rapidly emerging. Several studies have reported a significant spike in hospital admissions for IBD (both Crohn’s disease and ulcerative colitis) in the days and weeks following a heatwave [223, 224]. A study in Switzerland found that each additional hot day per week increased the risk of an IBD flare-related hospitalization by 4.6% [223]. This human-level data strongly supports the mechanistic “leaky gut” pathway, wherein heat-induced splanchnic vasoconstriction and subsequent ischemia compromise the intestinal barrier, leading to LPS translocation and a flare of underlying inflammation [225]. Once endotoxin crosses a damaged intestinal barrier, it can engage TLR4-dependent innate immune pathways, promote TNF-α, IL-1β and IL-6 release, injure endothelial and glycocalyx structures, and amplify systemic inflammation. Under severe heat stress or coexisting infection, this pathway may contribute to a feed-forward cycle of endotoxemia, microvascular leakage, organ injury and septic-shock-like physiology [226, 227]. Similar associations are found for infectious gastroenteritis, as warmer temperatures promote the replication of foodborne and waterborne pathogens (*Salmonella*, *Campylobacter*, etc.), placing an increased microbial load on a gut barrier that may already be weakened by thermal stress [228] (Fig. 6).

Climate change is also reshaping the epidemiology of vector-borne diseases, adding an infectious dimension to the temperature-driven health burden discussed above. Because the pathogens, arthropod vectors, and reservoir hosts that sustain these diseases are all highly temperature-sensitive, warming alters vector behavior, physiology, and life history, lengthens transmission seasons, and shifts the geographic range over which transmission can occur [229].

Malaria, the most studied climate-sensitive vector-borne disease, has spread to higher elevations in the Colombian and Ethiopian highlands in association with warming of roughly 0.2 °C per decade [230]. Dengue, the most common mosquito-borne viral infection with an estimated 390 million cases annually across more than 100 countries, continues to expand as its Aedes aegypti and A. albopictus vectors move into previously temperate regions, with autochthonous transmission now reported in parts of Europe; climate change is projected to become the dominant driver of this expansion by 2030 [231]. In parallel, warming winters and extended summers have driven the northward spread of Ixodes ticks into Canada and Norway with corresponding increases in Lyme disease, while West Nile virus, whose human incidence peaks near 24 °C, is shifting its transmission northward, including newly established local transmission in Germany after unusually warm weather [232, 233]. These shifts mean that climate-stressed populations face not only impaired epithelial barrier defenses but also an enlarging geographic and seasonal window of exposure to vector-borne pathogens, compounding the overall infectious and inflammatory burden.

Skin, the primary thermal interface, shows clear responses. ED visits for atopic dermatitis and contact dermatitis often increase with heat [234, 235]. Particularly in conditions of high humidity promote miliaria (heat rash) and skin maceration, providing an entry point for irritants and opportunistic skin bacteria [236] (Fig. 6).

Beyond the primary barriers, heat stress is a major risk factor for acute kidney injury and chronic kidney disease progression, as recurrent dehydration and rhabdomyolysis (in exertional heat stress) place an extreme burden on the kidneys [237]. Systemic inflammation from heat-triggered LPS translocation can acutely strain the cardiovascular system, raising the risk of myocardial infarction and stroke in susceptible groups [226, 227] (Fig. 6).

### Cold Exposure, infections and allergy

**Fig. 7:**
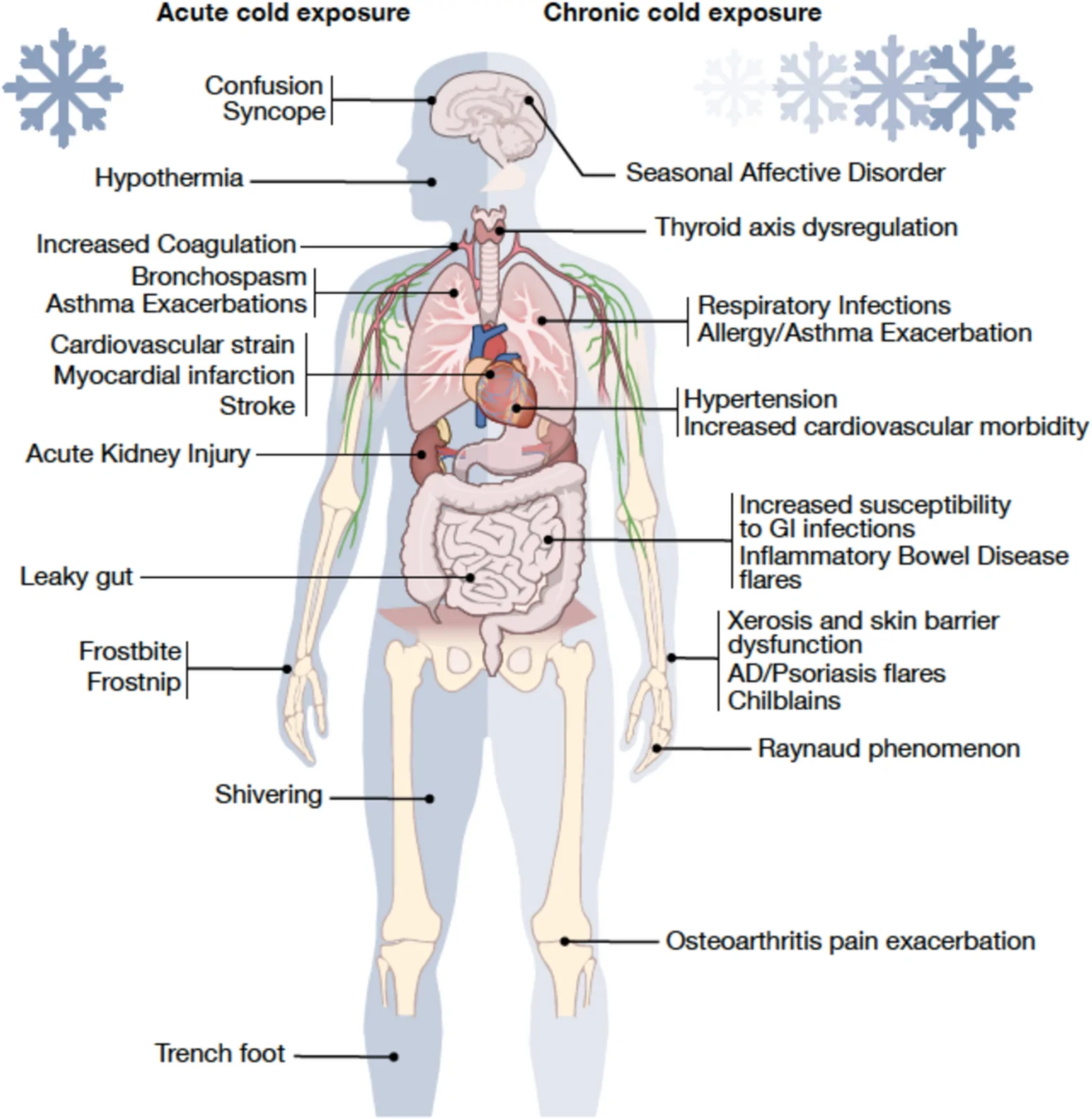
Systemic pathophysiology of acute versus chronic cold exposure. The left panel (acute cold exposure) shows immediate physiological complications, including neurological impacts (confusion, syncope), hypothermia, respiratory exacerbations (bronchospasm, asthma exacerbations), cardiovascular strain (increased coagulation, myocardial infarction, stroke), acute kidney injury, gastrointestinal permeabilization, musculoskeletal shivering, and direct tissue injuries, including frostbite, frostnip, and trench foot. The right panel (chronic cold exposure) outlines long-term disease progression and increased susceptibility. Key effects include neurological and endocrine dysregulation (seasonal affective disorder, thyroid axis dysregulation), respiratory vulnerabilities (infections, allergy/asthma exacerbation), cardiovascular morbidity (hypertension), gastrointestinal vulnerabilities (increased susceptibility to GI infections, inflammatory bowel disease flares), osteoarthritis pain exacerbation, and dermatological issues including xerosis, skin barrier dysfunction, AD/psoriasis flares, chilblains, and Raynaud phenomenon. AD, atopic dermatitis; GI, gastrointestinal

Epidemiological and immunological research confirms increased rates of infection and inflammation during cold weather, supporting common observations about illness in winter. Cold air directly facilitates respiratory infections and triggers non-infectious inflammatory NCDs (Fig. 7).

The seasonality of influenza, rhinoviruses, and coronaviruses is strongly linked to drops in temperature and, critically, absolute humidity [238]. Cold, dry air impairs the respiratory barrier’s *innate* immune functions. Cold, dry air diminishes mucociliary clearance, a vital process for trapping and removing inhaled pathogens [239]. Recent studies demonstrated that at 33–35 °C nasal temperatures, innate immune signaling and antiviral interferon production are depressed compared to 37 °C, favoring viral infection [240]. This creates a permissive window for viral replication. Therefore, cold does not “create” the virus, but it significantly degrades the epithelial barrier’s first line of defense, increasing susceptibility to infection.

For patients with asthma and COPD, cold air is a primary, non-allergic trigger for acute exacerbations. As discussed previously, the inhalation of cold, dry air induces osmotic stress and cooling of the airway epithelium, triggering mast cell degranulation and the release of inflammatory mediators (histamine, leukotrienes) that drive bronchoconstriction and inflammation. This mechanism is so reliable it is used diagnostically as the “eucapnic voluntary hyperpnea” challenge, which mimics the effect of cold-air exercise [241].

The link between winter and flares of AD and psoriasis is well-established. Epidemiological data show a clear seasonality, with symptom scores worsening as temperature and humidity drop [242]. This is directly attributable to barrier failure: cold, dry air increases transepidermal water loss, while cold-induced vasoconstriction reduces the delivery of nutrients for barrier repair [234]. This cold-induced xerosis (dry skin) compromises the skin barrier function, allowing for increased penetration of allergens and irritants, which can drive or exacerbate the underlying Type 2 immune-mediated inflammation characteristic of AD.

### Environmental changes, barrier breach, and infectious disease epidemiology

Barrier breach provides a direct mechanistic route by which thermal stress and altered weather patterns can increase the infection risks. Rather than simply letting subcellular inflammatory fragments drain through the epithelium and out of the tissues, a physically damaged mucosal barrier allows live obligate and opportunistic pathogens and commensals to cross directly between epithelial cells [214, 215] During extreme heatwaves or floods, the tight junctions sealing the gut are compromised. This allows enteric pathogens, such as Salmonella, Campylobacter, and Vibrio cholerae to breach the epithelial sheet and invade host tissues [228, 243]. Epidemiologically, this physical leakiness lowers the threshold for infection, suggesting that exposure to fewer numbers of pathogens can be required to make a patient clinically ill. This mechanism explains the clear seasonal and weather-related spikes seen in waterborne and foodborne gastroenteritis [223, 228]. Consequently, in a temperature-stressed host, what starts as simple surface colonization can rapidly progress to an invasive infection, bacteremia and systemic illness can occur without requiring any pre-existing immune deficiency. Once the intestinal barrier becomes permeable, lipopolysaccharide (LPS) and other microbial products can enter portal and systemic circulation. These molecules activate TLR4-dependent signaling in monocytes, macrophages, endothelial cells and epithelial cells, driving NF-κB activation, cytokine release, complement engagement, endothelial glycocalyx injury and coagulation pathway activation [226, 227]. This creates a continuum from transient endotoxemia to systemic inflammation, microvascular dysfunction, organ injury and septic-shock-like physiology, particularly when dehydration, exertional heat illness, infection, contaminated water exposure or pollutant co-exposure are present. This pathway is central to epithelial barrier theory because it links a physical environmental exposure to microbial translocation and immune dysregulation without requiring a primary invasive infection at the outset. The term septic-shock-like physiology should therefore not imply that every heat-exposed individual develops culture-positive sepsis, but rather that thermal injury can generate an endotoxin-driven inflammatory state that overlaps mechanistically with sepsis biology.

Altered weather patterns also reshape infectious-disease epidemiology by modifying pathogen survival, replication, environmental persistence, vector ecology, reservoir behavior and host exposure. Rising temperatures, shifting precipitation and extreme weather events can expand or redistribute ecological niches in which pathogens, vectors and reservoirs interact [244]. Vector-borne infections illustrate this principle because temperature affects mosquito development, biting frequency, survival and pathogen replication. Mosquito-borne pathogens such as dengue virus and Plasmodium falciparum show temperature-sensitive transmission dynamics, meaning that warming can shift the geographic and seasonal window of risk rather than simply increasing transmission everywhere [245]. Waterborne and foodborne infections are similarly affected. Extreme precipitation and flooding can overwhelm sewage and drinking-water systems, increasing exposure to enteric pathogens and microbial products that challenge the intestinal barrier [125, 126]. Cholera distribution has been linked to El Niño-associated hydrological variability, and flood-associated contamination can increase exposure to organisms such as Salmonella and Cryptosporidium [243]. In these settings, infection risk depends not only on pathogen abundance, but also on whether the gut barrier has already been weakened by heat stress, dehydration, malnutrition, inflammatory bowel disease, medication use or pollutant exposure.

The respiratory barrier is exposed to infectious and inflammatory bioaerosols after droughts, dust storms and floods. Dust storms can transport mineral dust, Gram-negative bacterial fragments, airborne endotoxins, fungal spores, pollen fragments, pesticide residues and adsorbed pollutants over long distances [130]. These mixed aerosols deposit across the nasal, bronchial and small-airway epithelium, where particle abrasion, osmotic stress and oxidative injury can disrupt ciliary activity and tight junction integrity. Endotoxin activates TLR4-dependent innate immune pathways and intensifies neutrophilic airway inflammation, while fungal spores and mold fragments can induce alarmin release, dendritic-cell activation and type 2 or mixed inflammatory responses. Floods and water-damaged buildings create a related exposure setting through sewage contamination, stagnant water, indoor dampness and damaged ventilation systems that aerosolize microbial products, mold spores and chemical residues (125, 126). Thunderstorm-associated asthma provides another barrier-relevant example, because pollen grains can rupture under osmotic shock and release respirable allergen-containing particles that reach the lower airways more efficiently than intact pollen grains [128]. These exposures may not always directly cause infection, but they weaken epithelial defenses, impair mucociliary clearance and create an inflammatory environment that favors viral, bacterial or fungal complications in susceptible individuals.

Zoonotic spillover adds another layer to this framework. Changes in temperature, precipitation, vegetation, food availability and habitat suitability can alter reservoir density, migration and human-animal contact. Modelling suggests that warming may increase cross-species viral sharing by reshaping mammalian range overlap [246]. Spillover and spillback dynamics, highlighted during the SARS-CoV-2 pandemic, further support a One Health interpretation that includes humans, domestic animals, wildlife and shared environments [247]. The 1990s hantavirus outbreak in North America illustrates how climate-associated food abundance increased deer-mouse populations and facilitated indirect human exposure to infected excreta, contributing to hantavirus pulmonary syndrome [248]. This example is relevant to epithelial barrier biology because inhaled viral particles encounter a respiratory surface whose defenses may already be altered by dust, smoke, cold air, dryness or other co-exposures.

Coccidioidomycosis, or Valley fever, provides a particularly relevant fungal example. Coccidioides immitis and Coccidioides posadasii live in soil, and their ecology is influenced by temperature, precipitation, drought, soil disturbance and aerosolization. Climate-suitability modelling projects expansion of endemic regions in the United States under warming scenarios, indicating that altered temperature and precipitation may increase the geographic area suitable for transmission [249]. Drought, dust storms and changing land-surface conditions may further increase airborne exposure to Coccidioides arthroconidia [250]. This creates a direct bridge between altered weather patterns and barrier exposure: the airway epithelium becomes the portal of entry for a pathogen whose range, seasonality and dispersal are shaped by heat, rainfall, drought and soil disturbance. Similar logic applies to other bacterial, fungal, viral and vector-borne infections. From the perspective of epithelial barrier theory, altered infectious-disease epidemiology is therefore not only a matter of pathogen distribution. It is also a matter of barrier timing. Heat, dehydration, smoke, dust, chemical exposure, cold-associated mucosal drying or floodwater contact may weaken the skin, gut or airway barrier before or during pathogen exposure, increasing the likelihood of colonization, invasion, sensitization or prolonged inflammation. This supports integrated surveillance combining climate data, air and water quality, occupational exposure, pathogen monitoring and barrier-focused clinical outcomes.

### Linking temperature extremes and altered weather patterns to NCD prevalence

We are, in effect, weakening our barriers from multiple angles simultaneously. A child’s respiratory epithelium, already dysregulated from reduced microbial education and chronically inflamed by urban PM2.5 exposure, is now subjected to more frequent and intense heatwaves that amplify ozone production and more volatile temperature swings that impair innate defenses. This multi-hit accumulation of barrier damage, over a lifetime, may lower the threshold for developing a clinical NCD. Epidemiological studies are beginning to support this framework, finding that children living in high-pollution areas who are *also* exposed to wildfire smoke and related environmental stressors have a synergistically higher risk of developing new-onset asthma [120].

This link is no longer theoretical. A 2024 global analysis quantifying the burden of NCDs attributable to high temperature from 1990 to 2019 confirms this trend. The study found that the global burden is substantial and increasing; in 2019 alone, high temperatures were responsible for approximately 150,000 deaths and 3.4 million disability-adjusted life years from NCDs [7]. This burden showed a clear upward trend globally between 1990 and 2019, with the number of deaths increasing more than six fold. Cardiovascular diseases, including stroke, were the primary contributors to this burden. The analysis also identified vulnerable populations, showing a higher burden in males, the elderly, and regions with lower socioeconomic development, such as South Asia, North Africa, and the Middle East [7].

This framework highlights that the health implications of climate change are not just about acute events but about the long-term erosion of our fundamental physiological resilience, with epithelial barrier integrity at its core.

## Conclusion

Extreme temperature exposure represents an emerging yet insufficiently recognized driver of epithelial barrier dysfunction, with broad impact on global health. The evidence reviewed here demonstrates that both heat and cold exert profound stress on epithelial surfaces, compromising structural integrity, altering tight-junction regulation and reshaping local immune responses. These perturbations weaken barrier defenses and increase susceptibility to infections, allergic sensitization and chronic NCDs. The mechanistic pathways, including tight-junction disruption, oxidative stress, endoplasmic reticulum stress, mitochondrial dysfunction, increased apoptosis and temperature-induced cytokine shifts, illustrate that temperature extremes act as potent physiological disruptors. It is essential to acknowledge that temperature does not exert its effects independently. Rather, complex interactions among environmental factors, such as air pollution, UV radiation, humidity and climate-driven events, collectively intensify exposure, thereby exacerbating stress on the epithelial barrier. Environmental disasters such as wildfires and floods markedly elevate the burden of airborne particulates, microbial-derived toxins, waterborne contaminants and chemical pollutants. The increased exposure to these diverse hazards acts synergistically to undermine epithelial barrier integrity. Clinical and epidemiological data now consistently show correlations between extreme temperatures and disease morbidity. With global temperatures continuing to rise, protecting epithelial barrier integrity should be recognized as an essential public health priority. Progress in this area will require the combined application of high-resolution exposome profiling, mechanistic studies and real-world epidemiological monitoring to elucidate the links between extreme temperatures and diseases fully. Efforts to support epithelial barrier function may provide an effective strategy to mitigate the health impacts of climate instability.

## Data Availability

No datasets were generated or analysed during the current study.
